# Supplementary material for: Short-Term Dietary Restriction Potentiates an Anti-Inflammatory Circulating Mucosal-Associated Invariant T-Cell Response
Source: Nutrients. 2024 Apr 22;16(8):1245. doi: 10.3390/nu16081245 (PMC11053749; doi:10.3390/nu16081245)
Supplement: Supplementary file 1 [file nutrients-16-01245-s001.zip › nutrients-2944695-Supplementary Tables and Figures.pdf]

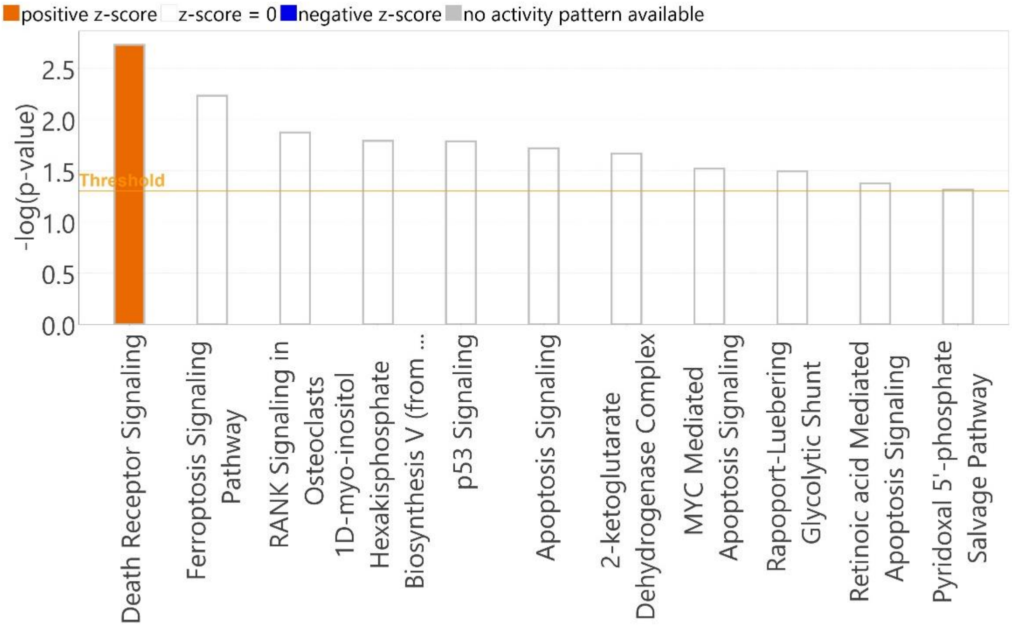

Figure S1. All enriched canonical pathways on day 7 compared to baseline.

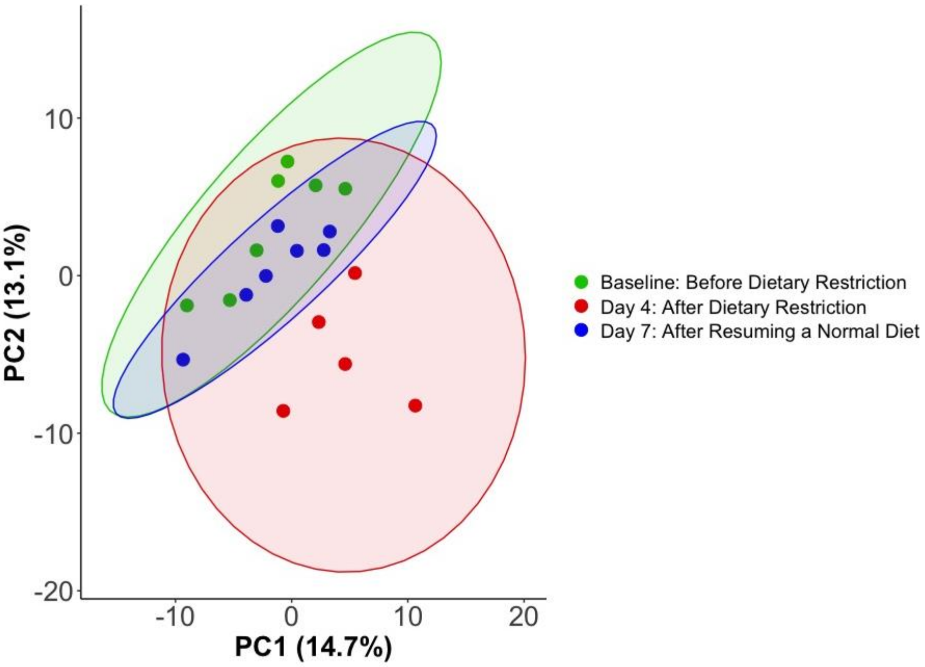

(**A**)

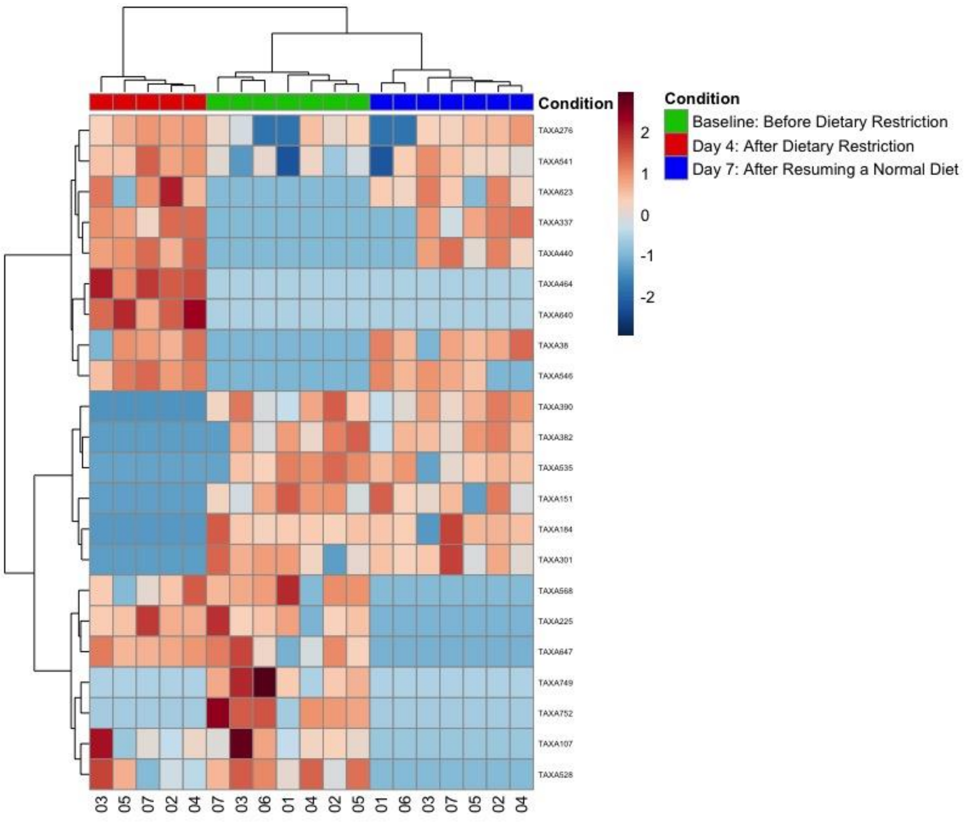

(**B**)

Figure S2. Principal component analysis and heatmap of species abundance after prevalence filtering. (**A**) PCA; (**B**) Heatmap.

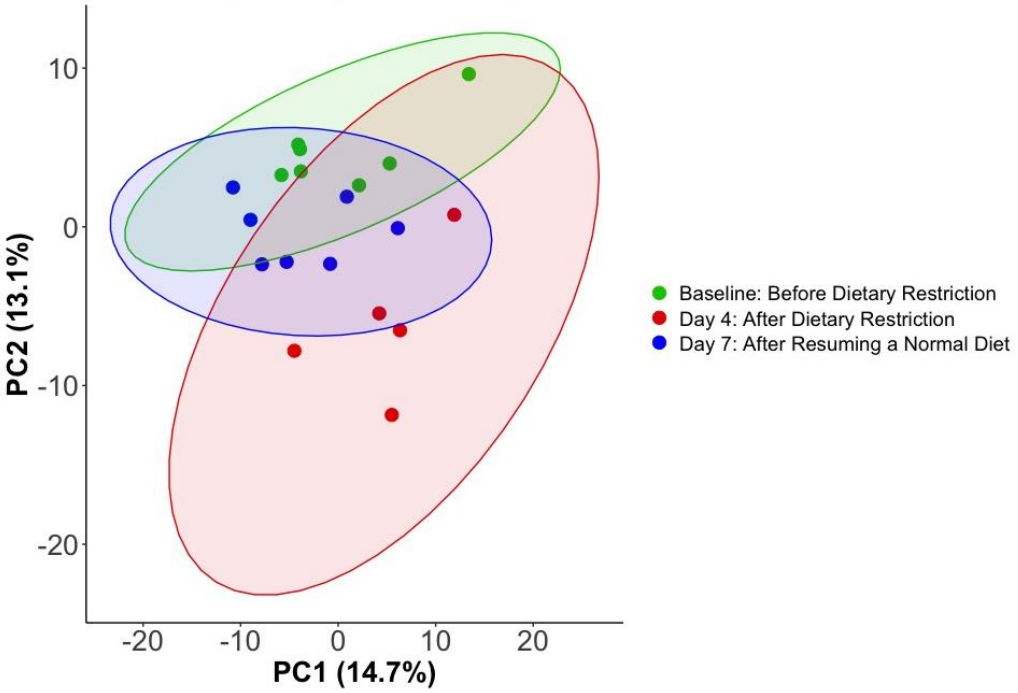

Figure S3. Principal component analysis of metabolic pathway changes after prevalence filtering.

Table S1. The top 20 differentially expressed genes for each condition compared to baseline. Genes are ordered from the most upregulated (rows 1–10) to the most downregulated (rows 11–20). The log-fold change of each gene is displayed in the "LogFC" column.

	**Day 2 Genes**		**Day 4 Genes**		**Day 7 Genes**	
Row ID	Gene ID	LogFC	Gene ID	LogFC	Gene ID	LogFC
1	ZC4H2	0.83	HSPA1B	0.72	ARID5B	0.68
2	FAM135A	0.67	KCNQ5	0.60	NLRC5	0.67
3	STX18-AS1	0.64	HIBADH	0.54	STX18-AS1	0.65
4	NLRC5	0.63	CHCHD3	0.54	FAM135A	0.62
5	ALG9	0.59	HSPA1A	0.52	RALGAPA2	0.62
6	LINC01422	0.59	FAM135A	0.51	STK38L	0.60
7	KCNQ5	0.55	SRGAP2	0.51	MMS22L	0.60
8	CD84	0.53	PCCB	0.51	AL359232.1	0.58
9	ZNF519	0.53	HDAC9	0.51	IFI44L	0.57
10	IFNG-AS1	0.51	STX6	0.49	ORC5	0.57
11	BTG2	−0.87	ZFP36	−0.74	ARRDC3	−0.86
12	NFKBIA	−0.83	ID1	−0.69	RSRC2	−0.70
13	ZFP36	−0.80	ACSL3	−0.69	ERCC1	−0.63
14	SLC7A5	−0.79	SLC7A5	−0.67	AL021918.5	−0.61
15	PIK3IP1	−0.74	DCTN4	−0.66	CEP19	−0.55
16	GADD45B	−0.73	SMAD7	−0.66	DCTN4	−0.54
17	FOS	−0.73	PRKX	−0.64	RPAP2	−0.54
18	IER5	−0.73	PMAIP1	−0.64	MMP24OS	−0.53
19	AC020916.1	−0.71	PDE4B	−0.64	NDUFB1	−0.52
20	KDM6B	−0.67	NFKBIA	−0.62	EFCAB2	−0.52
